# RETRACTION: Evaluating the Efficacy of Standardized Pressure Ulcer Management Protocols in the Prevention of Pressure Injuries Among Patients Undergoing Neurosurgical Procedures

**DOI:** 10.1111/iwj.70500

**Published:** 2025-04-02

## Abstract

**RETRACTION:** ZhangJ. , LiW. , LiY. , MaM. , and ShangK. , “Evaluating the Efficacy of Standardized Pressure Ulcer Management Protocols in the Prevention of Pressure Injuries Among Patients Undergoing Neurosurgical Procedures,” International Wound Journal 21, no. 4 (2024): e14879, 10.1111/iwj.14879.38581264 PMC10998278

The above article, published online on 06 April 2024, in Wiley Online Library (http://onlinelibrary.wiley.com/), has been retracted by agreement between the journal Editor in Chief, Professor Keith Harding; and John Wiley & Sons Ltd. Following an investigation by the publisher, all parties have concluded that this article was accepted solely on the basis of a compromised peer review process. The editors have therefore decided to retract the article. The authors did not respond to our notice regarding the retraction.

**RETRACTION:** J. Zhang , W. Li , Y. Li , M. Ma , and K. Shang , “Evaluating the Efficacy of Standardized Pressure Ulcer Management Protocols in the Prevention of Pressure Injuries Among Patients Undergoing Neurosurgical Procedures,” International Wound Journal 21, no. 4 (2024): e14879, 10.1111/iwj.14879.38581264 PMC10998278

The above article, published online on 06 April 2024, in Wiley Online Library (http://onlinelibrary.wiley.com/), has been retracted by agreement between the journal Editor in Chief, Professor Keith Harding; and John Wiley & Sons Ltd. Following an investigation by the publisher, all parties have concluded that this article was accepted solely on the basis of a compromised peer review process. The editors have therefore decided to retract the article. The authors did not respond to our notice regarding the retraction.

